# Optimal placement of tsunami sensors with depth constraint

**DOI:** 10.7717/peerj-cs.685

**Published:** 2021-09-29

**Authors:** Ikha Magdalena, Raynaldi La’lang, Renier Mendoza, Jose Ernie Lope

**Affiliations:** 1 Faculty of Mathematics and Natural Sciences, Institut Teknologi Bandung, Bandung, West Java, Indonesia; 2 Institute of Mathematics, University of the Philippines Diliman, Quezon City, Philippines

**Keywords:** Shallow water equations, Near-shore detection sensors, Particle swarm optimization

## Abstract

Tsunamis are destructive natural disasters that can cause severe damage to property and the loss of many lives. To mitigate the damage and casualties, tsunami warning systems are implemented in coastal areas, especially in locations with high seismic activity. This study presents a method to identify the placement of near-shore detection sensors by minimizing the tsunami detection time, obtained by solving the two-dimensional shallow water equations (SWE). Several benchmark tests were done to establish the robustness of the SWE model, which is solved using a staggered finite volume method. The optimization problem is solved using particle swarm optimization (PSO). The proposed method is applied to different test problems. As an application, the method is used to find the optimal location of a detection sensor using data from the 2018 Palu tsunami. Our findings show that detection time can be significantly reduced through the strategic placement of tsunami sensors.

## Introduction

Tsunami waves are, in most cases, generated by a seabed deformation caused by some tectonic plate movement, which leads to a sudden displacement of water that travels from the ocean to the shore. These waves may also be generated by landslides, submarine volcanic eruptions, and meteorological disturbances (Joseph, 2011; Okal & Synolakis, 2015). Unlike tidal waves, they characteristically have longer wavelength and amplitudes that start relatively small but builds up and undergoes shoaling as they reach shallower areas.

In the past 5 years alone, there have been five major tsunami occurrences: in 2016 in Kaikoura, New Zealand (Heidarzadeh & Satake, 2017), in 2017 in Greenland (Chao et al., 2018), in 2018 in Sulawesi, Indonesia (Heidarzadeh, Muhari & Wijanarto, 2018), in 2019 in Sunda Strait, Indonesia (Grilli et al., 2019), and in 2020 in the Aegean Sea (Triantafyllou et al., 2021). The most devastating was the earthquake-triggered-with-underwater-landslide tsunami in Sulawesi, which killed more than 1,000 and injured over 600. These regions are among those with the highest tsunami risk due to their proximity to tectonic plate boundaries. To minimize the damage and casualties of tsunamis in these high-risk areas, it is necessary to have carefully designed countermeasures, such as tsunami warning systems.

Seismic-centered tsunami warning systems typically work in three stages (UN-ESCAP, 2009). As the seismic network detects seismic waves, it sends a signal to seismologists in the warning center. This data is then analyzed to assess whether or not the seismic waves have the potential to generate a tsunami, including the threat level. The responsible party then issues the warning to the public and decides on which action to take. While the exact chain of actions may differ from one country to another, it still follows these general steps (Murjaya, 2012; Doi, 2003).

An example of a tsunami warning system is the Deep-ocean Assessment and Reporting of Tsunamis (DART) (Mungov, Eble & Bouchard, 2012; Bernard & Meinig, 2011; Paros et al., 2011). It comprises three parts: bottom pressure recorders (BPRs), surface buoys, and satellites. Each BPR is anchored to the seafloor to record barometric pressure and temperature. The data are then logged to the buoy as the BPRs read the average water level. Finally, the data are transmitted to the warning center by satellite. Another type of tsunami warning system are near-shore gauges that are used in alerting high-risk coastal communities in case of local tsunamis (Satake, 2014; E2S Warning Signals, 2021). These systems include wave gauges that use ultrasonic waves and buoy and measure offshore sea levels at water depths of 50 to 200 m (Satake, 2014).

Numerous studies on tsunami warning systems have been undertaken by researchers from countries located in the Pacific ring of fire. Mulia & Satake (2020) present the evolution of past to present tsunami observing systems available in Japan. The optimal placement of sensors in Korea was studied by Lee, Jung & Shin, 2020a, Lee, Jung & Shin 2020b. Meanwhile, the use of the rainfall optimization algorithm in the placement of sensors in the Cotabato Trench, Philippines was investigated by Ferrolino, Lope & Mendoza (2020), and an integrated tsunami forecast and warning system, called SIPAT, has been developed and proven successful in Chile (Catalan et al., 2020).

A warning system’s performance depends on the ability to optimize the function of each subsystem, and there are many aspects to look at. There have also been numerical methods developed to reproduce the tsunami generation and propagation (Behrens, 2010; Cecioni et al., 2014). Wang & Li (2008) introduced a tsunami warning system that utilizes remote sensing and geographical information systems in monitoring, forecasting, detection, loss evaluation, and relief management. Mulia et al. (2020) proposed an enhanced detection system using airborne platforms, and a 3D topology design for underwater sensor networks was proposed by Lohan, Dube & Agrawal (2020).

Several researchers have proposed methods of optimizing the location of buoys and gauges. Lee, Jung & Shin (2020a) used the Cornell Multi-grid Coupled Tsunami (COMCOT) numerical model and a probabilistic approach to gauge the optimal region for tsunami detection instruments in the eastern sea of Korea. Meza, Catalán and Tsushima (2019) implemented an inversion algorithm to determine the optimal array configuration of offshore tsunami sensors for near-field tsunami forecasting based on three tsunami parameters: arrival time, maximum tsunami amplitude, and forecast skill. Navarrete et al. (2020) used empirical orthogonal function analysis together with a heuristic optimization technique to find the optimal locations of a network of tsunameters. Recently, Wu, Chen & Ghattas (2021) presented a fast and scalable computational framework for finding sensor locations to maximize the expected information gain for a predicted quantity of interest. These results, however, are not quite general as they are calibrated to fit specific bathymetric profiles, tsunami characteristics, or detection sensors. Ferrolino et al. (2020) used two-dimensional shallow water equations (SWE) to compute the travel time of tsunami waves and particle swarm optimization (PSO) to find the optimal placement of tsunami sensors. The waves were made to propagate over a wet bed in their simulations, and no particular constraints were specified. However, in real-life scenarios, several restrictions must be satisfied in configuring the detection sensors. For example, near-shore warning systems must be installed in the shallower portion of the sea. To make the system cost-effective, the gauge must be placed within a prescribed water depth and on locations where the tsunami can be detected at the earliest possible time.

The objective of this study is to address the limitations in the simulations done by Ferrolino et al. (2020). First, we use nonlinear SWE with a wet-dry procedure in modeling the tsunami wave propagation so that the model also applies to a water domain with islands. Second, we modify the optimization problem that calculates the optimal placement of sensors by adding a constraint on water depth. This can be used in finding the strategic locations of near-shore tsunami sensors. For example, in the Philippines, tsunami early warning systems are installed near the shore (DOST, DOST-PHILVOLCS, DOST-ASTI, 2021).

The numerical solution to the two-dimensional SWE was obtained using the finite volume method (FVM) on a staggered grid, which has been shown to be robust, accurate, and inexpensive (Pudjaprasetya & Magdalena, 2014; Magdalena, Rif’atin & Reeve, 2020; Magdalena, Erwina & Pudjaprasetya, 2015). The arising sensor location problem is a nonlinear programming because of the constraints. We use a penalty method to transform the nonlinear programming problem into an unconstrained optimization problem for this work. Since the resulting cost function is not smooth, we employ PSO, an evolutionary optimization algorithm that does not require the computation of gradients. It was initially formulated to simulate social behavior in colonies (Kennedy & Eberhart, 1995), but has since then been explored to solve problems in broader fields, including product design and manufacturing (Zhou et al., 2006), smart antenna (Wagih, Elkamchouchi & Lazinica, 2009), and of course tsunami detection (Ferrolino et al., 2020).

Following this introductory section are three parts. The following section discusses the numerical solution of the SWE using FVM on a staggered grid and the constrained optimization problem. The third section showcases the robustness and accuracy of the proposed algorithm to solve the fluid dynamics model numerically. The section also features our implementation of the PSO algorithm to solve the problem of tsunami detection, first applied to several test problems and finally to actual data obtained from the 2018 Palu tsunami incident. The final section contains our concluding remarks.

## Methods

### Fluid dynamics model

This section discusses the SWE, one of many models in fluid dynamics that can be used to model fluid motions. Due to their required assumptions, they are especially accurate in solving problems involving the ocean (Briganti & Dodd, 2009; Brocchini & Dodd, 2008; Le Roux, Staniforth & Lin 1998). They can also be used to simulate different tsunami waves, as done by Geyer & Quirchmayr (2017) and Zergani, Aziz & Viswanathan (2015). The SWE arise from a special case of the Navier–Stokes equations, which describe the conservation of mass and momentum. In the case of the SWE, the wavelength of the disturbance is assumed to be long, relative to the depth of the flow. Due to this assumption, the horizontal velocity along a vertical column can be assumed to be uniform, hence it is only a function of the horizontal spatial parameters, namely *x* and *y*.

#### Shallow water equations

**Figure 1:**
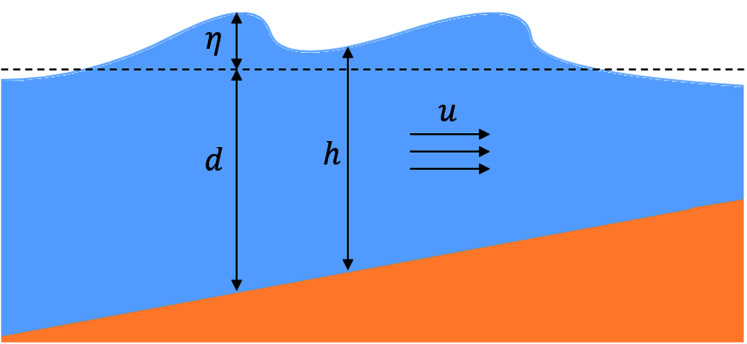
Illustration of variables used in the 1D SWE.

The equations in one dimension are obtained by taking a vertical cross-section (in this case, a *y*-plane cross-section) of some two-dimensional domain and neglecting flows not parallel to the cross-section. Assuming uniform fluid density, incompressible-irrotational flow, and frictionless bed, the set of equations in one dimension is

(1) $${h_t} + {(hu)_x} = 0,$$

(2) $${u_t} + g{\eta _x} + u{u_x} = 0,$$

where *h* is the total water depth, *u* is the horizontal velocity, and *g* is the gravitational acceleration (throughout this study, the value of *g* is set to 9.81 m.s^−2^). We also use the variables *d* for water depth and *η* for the free surface elevation, giving the relation *h* = *d* + *η*. Here, Eq. (1) represents the conservation of mass, while Eq. (2) represents the momentum balance. Figure 1 illustrates the variables just described.

To extend the model to two dimensions, we will have two equations for momentum balance in *x*- and *y*-direction. We would then need one more variable: *v* to denote the horizontal velocity in the *y*-direction. We also introduce the advection terms *vu*_*y*_ and *uv*_*x*_ in the momentum-conservation-equations. Thus the 2D governing equations read as:

(3) $${h_t} + {(hu)_x} + {(hv)_y} = 0,$$

(4) $${u_t} + g{\eta _x} + u{u_x} + v{u_y} = 0,$$

(5) $${v_t} + g{\eta _y} + v{v_y} + u{v_x} = 0.$$

**Figure 2:**
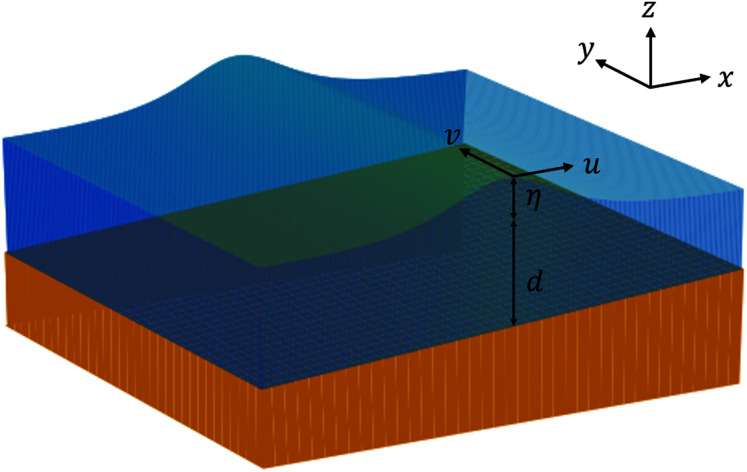
Illustration of variables used in the 2D SWE.

All the variables used in the two-dimensional SWE are illustrated in Fig. 2.

#### Finite volume method on a staggered grid

**Figure 3:**
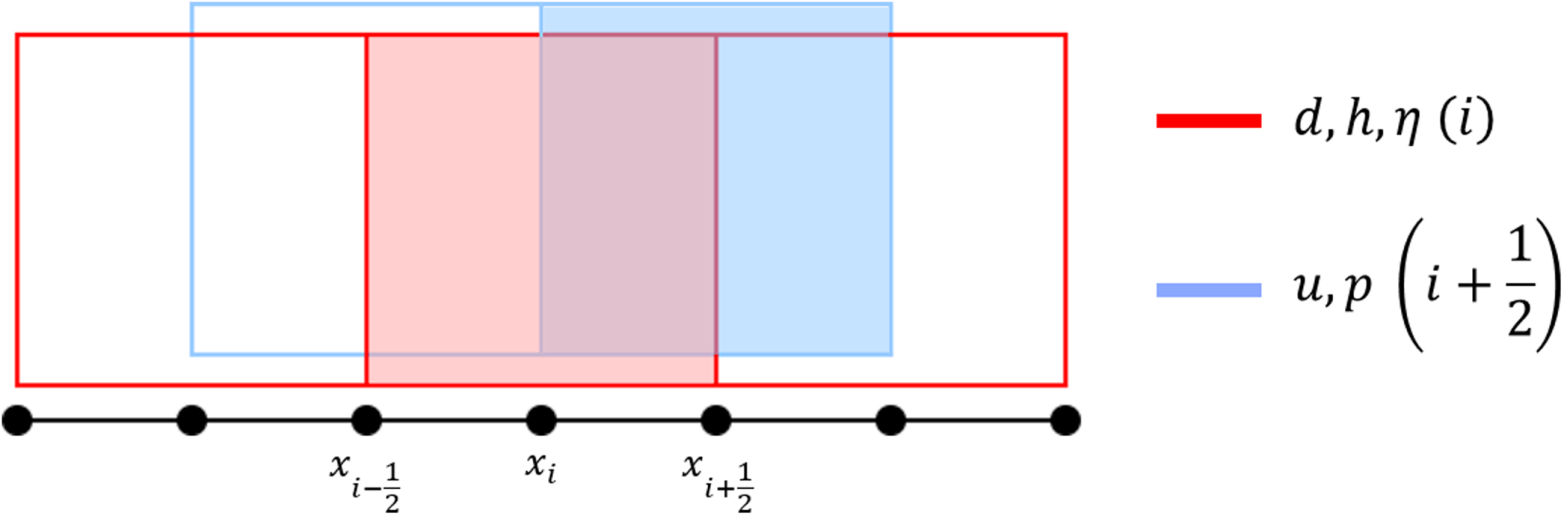
Staggered grid for the FVM scheme on the 1D SWE.

**Figure 4:**
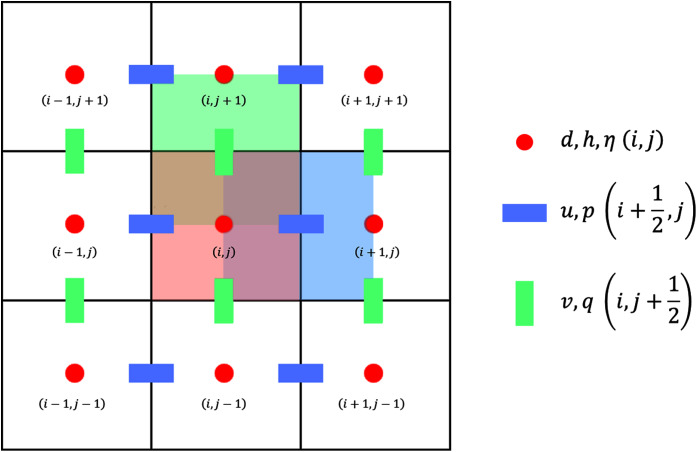
Staggered grid for the FVM scheme on the 2D SWE.

We apply the finite volume method on a staggered grid to solve the SWE numerically. In a staggered grid arrangement, *η* is stored in the cell centre, while vectors *u* and *v* are stored in the cell faces. We use a structured rectangular grid to partition the spatial domain into intervals of equal size *Δx* in one dimension, or rectangles of equal size *Δx* × *Δy* in two dimensions; integer indices (*x*_*i*_, *y*_*j*_) represent the cell centers while half-integer indices $({x_{i + \textstyle{1 \over 2}}},{y_{j + \textstyle{1 \over 2}}})$ represent the cell faces. These are visualized in Figs. 3 and 4. We denote by *t*_*n*_ the discretization of the time dimension.

Note that computing the spatial derivatives of *p* = *hu* and *q* = *hv* is not as straightforward, as they are products of two variables stored using two different indices. We approximate the values using the following upwind scheme to compute *h* at the cell faces, based on the direction of the flow:

(6) $$*{h_{i + \textstyle{1 \over 2}}} = \left\{ {\matrix{ {{h_i},}  {{u_{i + \textstyle{1 \over 2}}} \gt 0,} \cr {{h_{i + 1}},}  {{u_{i + \textstyle{1 \over 2}}} \le 0.}}} \right.}}$$

We then define $*{p_{i + \textstyle{1 \over 2}}} = *{h_{i + \textstyle{1 \over 2}}}{u_{i + \textstyle{1 \over 2}}}$. Now, to compute $\left( {u{u_x}} \right)_{i + \textstyle{1 \over 2}}^n$, we take note of the following equality:

(7) $$u{u_x} = \displaystyle{p \over h}{u_x}.$$

This is a modification of the method earlier used by Magdalena, Erwina & Pudjaprasetya (2015) and is preferred here because the calculation of the advection term is simpler and less costly. Next, the value of *h* at half-integer indices and the value of *p* and *u* at integer indices are computed as

(8) $${\overline h _{i + \textstyle{1 \over 2}}} = \textstyle{1 \over 2}\left( {{h_i} + {h_{i + 1}}} \right),$$

(9) $${\overline p _i} = \textstyle{1 \over 2}\left( {{p_{i + \textstyle{1 \over 2}}} + {p_{i - \textstyle{1 \over 2}}}} \right),$$

(10) $$*{u_i} = \left\{ {\matrix{ {{u_{i - \textstyle{1 \over 2}}},}  {{{\overline p }_i} \gt 0} \cr {{u_{i + \textstyle{1 \over 2}}},}  {{{\overline p }_i} \le 0.} } } \right.$$

These quantities yield the following approximation of $(u{u_x})_{i + \textstyle{1 \over 2}}^n$:

(11) $${\left( {u{u_x}} \right)_{i + \textstyle{1 \over 2}}} = \displaystyle{{{{\overline p }_{i + \textstyle{1 \over 2}}}} \over {{{\overline h }_{i + \textstyle{1 \over 2}}}\;}}\left( {\displaystyle{{*{u_{i + 1}} - *{u_i}} \over {\Delta x}}\;} \right),$$

Thus, we obtain this discretization of the one-dimensional SWE:

(12) $$\displaystyle{{h_i^{n + 1} - h_i^n} \over {\Delta t}} + \displaystyle{{*p_{i + \textstyle{1 \over 2}}^n - *p_{i - \textstyle{1 \over 2}}^n} \over {\Delta x}} = 0,$$

(13) $$\displaystyle{{u_{i + \textstyle{1 \over 2}}^{n + 1} - u_{i + \textstyle{1 \over 2}}^n} \over {\Delta t}} + g\displaystyle{{\eta _{i + 1}^{n + 1} - \eta _i^{n + 1}} \over {\Delta x}} + \left( {u{u_x}} \right)_{i + \textstyle{1 \over 2}}^n = 0.$$

Extending the finite volume method on SWE to two dimensions require us to compute $\left( {v{u_y}} \right)_{i + \textstyle{1 \over 2},j}^n$ $\left( {v{v_y}} \right)_{i,j + \textstyle{1 \over 2}}^n$ and $\left( {u{v_x}} \right)_{i,j + \textstyle{1 \over 2}}^n$. We only give the details in approximating the first expression, as the others can be done similarly. This time, we use the equalities

(14) $$v{u_y} = \displaystyle{q \over h}{u_y},\quad \quad v{v_y} = \displaystyle{q \over h}{v_y},\quad \quad and\quad \quad u{v_x} = \displaystyle{p \over h}{v_x}.$$

Analogous to the first advection term, we do an approximation for the following variables:

(15) $${\overline h _{i + \textstyle{1 \over 2},j}} = \textstyle{1 \over 2}\left( {{h_{i,j}} + {h_{i + 1,j}}} \right),$$

(16) $${\overline q _{i + \textstyle{1 \over 2},j + \textstyle{1 \over 2}}} = \textstyle{1 \over 2}\left( {{q_{i + 1,j + \textstyle{1 \over 2}}} + {q_{i,j + \textstyle{1 \over 2}}}} \right),$$

(17) $$*{u_{i + \textstyle{1 \over 2},j + \textstyle{1 \over 2}}} = \left\{ {\matrix{ {{u_{i + \textstyle{1 \over 2},j}},}  {{{\overline q }_{i + \textstyle{1 \over 2},j + \textstyle{1 \over 2}}} \gt 0} \cr {{u_{i + \textstyle{1 \over 2},j + 1}},}  {{{\overline q }_{i + \textstyle{1 \over 2},j + \textstyle{1 \over 2}}} < 0.} \cr } } \right.$$

Thus, we have these approximations:

(18) $${\left( {v{u_y}} \right)_{i + \textstyle{1 \over 2},j}} = \displaystyle{{{{\overline q }_{i + \textstyle{1 \over 2},j}}} \over {{{\overline h }_{i + \textstyle{1 \over 2},j}}\;}}\left( {\displaystyle{{*{u_{i + \textstyle{1 \over 2},j + \textstyle{1 \over 2}}} - *{u_{i + \textstyle{1 \over 2},j - \textstyle{1 \over 2}}}} \over {\Delta y}}} \right),$$

(19) $${\left( {v{v_y}} \right)_{i,j + \textstyle{1 \over 2}}} = \displaystyle{{{{\overline q }_{i,j + \textstyle{1 \over 2}}}} \over {{{\overline h }_{i,j + \textstyle{1 \over 2}}}\;}}\left( {\displaystyle{{{v_{i,j + \textstyle{1 \over 2}}} - {v_{i,j - \textstyle{1 \over 2}}}} \over {\Delta y}}} \right),$$

(20) $${\left( {u{v_x}} \right)_{i,j + \textstyle{1 \over 2}}} = \displaystyle{{{{\overline p }_{i,j + \textstyle{1 \over 2}}}} \over {{{\overline h }_{i,j + \textstyle{1 \over 2}}}\;}}\left( {\displaystyle{{*{v_{i + \textstyle{1 \over 2},j + \textstyle{1 \over 2}}} - *{v_{i + \textstyle{1 \over 2},j - \textstyle{1 \over 2}}}} \over {\Delta x}}} \right),$$

and, finally, we obtain this discretization of the two-dimensional SWE:

(21) $$\displaystyle{{h_{i,j}^{n + 1} - h_{i,j}^n} \over {\Delta t}} + \displaystyle{{{\;^*}p_{i + \textstyle{1 \over 2},j}^n - {\;^*}p_{i - \textstyle{1 \over 2},j}^n} \over {\Delta x}} + \displaystyle{{{\;^*}q_{i,j + \textstyle{1 \over 2}}^n - {\;^*}q_{i,j - \textstyle{1 \over 2}}^n} \over {\Delta y}} = 0,$$

(22) $$\displaystyle{{u_{i + \textstyle{1 \over 2},j}^{n + 1} - u_{i + \textstyle{1 \over 2},j}^n} \over {\Delta t}} + g\displaystyle{{\eta _{i + 1,j}^{n + 1} - \eta _{i,j}^{n + 1}} \over {\Delta x}} + \left( {u{u_x}} \right)_{i + \textstyle{1 \over 2},j}^n + \left( {v{u_y}} \right)_{i + \textstyle{1 \over 2},j}^n = 0,$$

(23) $$\displaystyle{{v_{i,j + \textstyle{1 \over 2}}^{n + 1} - v_{i,j + \textstyle{1 \over 2}}^n} \over {\Delta t}} + g\displaystyle{{\eta _{i,j + 1}^{n + 1} - \eta _{i,j}^{n + 1}} \over {\Delta y}} + \left( {v{v_y}} \right)_{i,j + \textstyle{1 \over 2}}^n + \left( {u{v_x}} \right)_{i,j + \textstyle{1 \over 2}}^n = 0.$$

### Proposed approach in Tsunami sensor placement

The sensors should be placed in locations where detection time is minimized. We now discuss the optimization formulation arising from this real-world application. We modify the minimization problem proposed by Ferrolino et al. (2020), Ferrolino, Lope & Mendoza (2020) by adding a constraint on the water depth.

#### Optimization problem

Suppose we have source points $R = \{ {r_1}, \ldots ,{r_k}\}$, where tsunami waves are generated. Let *s* be an arbitrary point in the spatial domain. We define

(24) $$\eqalign{\tau \left( {{r_i};s} \right): = {\rm the\ time\ it\ takes\ for\ a\ wave\ generated\ from\ a\ source\ point } \cr\quad {r_i\ {\rm to\ reach\ the\ point\ {\it s.}}}}$$

To compute $\tau \left( {{r_i};s} \right)$, we use the two-dimensional SWE presented previously. Suppose we wish to place *l* sensors, $\sigma = \{ {s_1}, \ldots ,{s_l}\}$. Since only one sensor is needed to be triggered for the tsunami to detected, the time it takes for the tsunami to be detected from a source *r*_*i*_ is given by

$$t({r_i};\sigma ): = \mathop {\min }\limits_{1 \le j \le l} \tau \left( {{r_i};{s_j}} \right).$$

Thus, the guaranteed tsunami detection time to reach at least one of the sensors $\sigma = \{ {s_1}, \ldots ,{s_l}\}$ from any of the possible sources is given by

(25) $$T(\sigma ) = \mathop {\max }\limits_{1 \le i \le k} t({r_i};\sigma ).$$

Suppose the sensors can be placed anywhere in the water domain *D*, then the goal is to minimize (25). This minimization problem was also used in previous studies (Ferrolino, Lope & Mendoza, 2020, Ferrolino et al., 2020; Astrakova et al., 2009).

**Figure 5:**
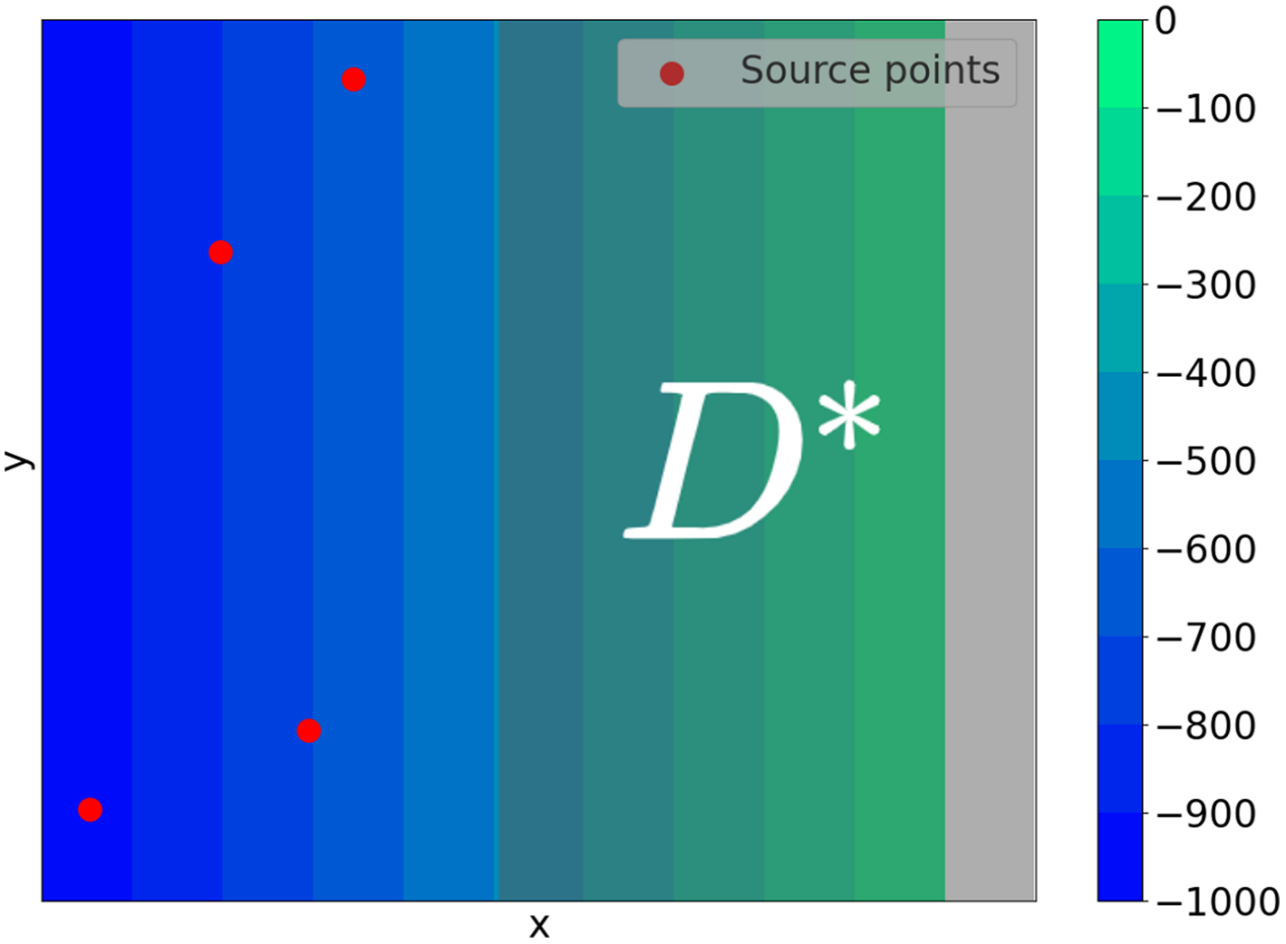
This is an example of a domain *D*. The tsunami sources are shown in red and the set of points that are within the depth constraint is labeled D*.

In this work, we introduce a constraint by considering the scenario where the sensors can only be placed on locations with depth constraint, as in the case of near-shore tsunami sensors. If we denote by D* the points in *D* that are within the depth constraint (see Fig. 5), then the minimization problem we consider is given by

(26) $$\left\{ {\matrix{ {\mathop {\min }\limits_\sigma {\kern 1pt} {\kern 1pt} T(\sigma ),} \cr {{\rm subject\ to}\ \sigma \in {D^*}.}} } \right.$$

The constrained domain D* can be defined as D* = {(*x*, *y*) ∈ *D*: *d*(*x*, *y*) ≤ *d*_*max*_ }, where *d*_*max*_ is the maximum depth in which a sensor can be placed. The constrained optimization problem (26) can be reformulated as an unconstrained problem using the penalty method, that is, by considering the problem

(27) $$\mathop {\min }\limits_\sigma T(\sigma ) + \mu {\chi _{D\backslash {D^*}}}(\sigma ),$$

where *μ* is the penalty parameter and ${\chi _{D\backslash {D^{*}}}}$ (*σ*) is the characteristic function (or indicator function) of *D*\D*, whose value is 0 if *σ* ∈ D* and 1 otherwise. We set *μ* equal to a large number to penalize those sensor locations that do not satisfy the depth constraint. In our simulations, we set *μ* = 10^6^.

It should be noted that this formulation can be easily modified to consider cases when the tsunami does not originate from a point source (*e.g*., when the tsunami is due to a landslide). The minimization problem remains the same but the initial conditions of the SWE will have to be modified. Likewise, this approach can also be used to determine the location of deep-ocean tsunami sensors by either setting the depth constraint to a high value or the value of *μ* to 0.

#### Particle swarm optimization

As discussed previously, the function *τ* in (24) is computed by solving the two-dimensional SWE numerically. However, this only gives us the values of *τ* at points located at the cell centers of the rectangular grid. To calculate the travel time at other locations, we use bilinear interpolation using the values of *τ* at the surrounding points.

Bilinear interpolation is done by performing linear interpolation in one direction, and then performing it again in the other direction, on every rectangle [*x*_*i*_, *x*_*i*__+ 1_] × [*y*_*j*_, *y*_*j*__+ 1_]. For example, suppose we want to interpolate *τ* using some function *f*(*x*, *y*) on the unit square [0, 1] × [0, 1], given its values at the points (0, 0), (1, 0), (0, 1), and (1, 1). Then *f*(*x*, *y*) is given by

(28) $$\eqalign{ f(x,y) = f(0,0)(1 - x)(1 - y) + f(1,0)x(1 - y) + f(0,1)(1 - x)y + f(1,1)xy, \cr\quad (x,y) \in {[0,1]^2.}}$$

**Figure 6:**
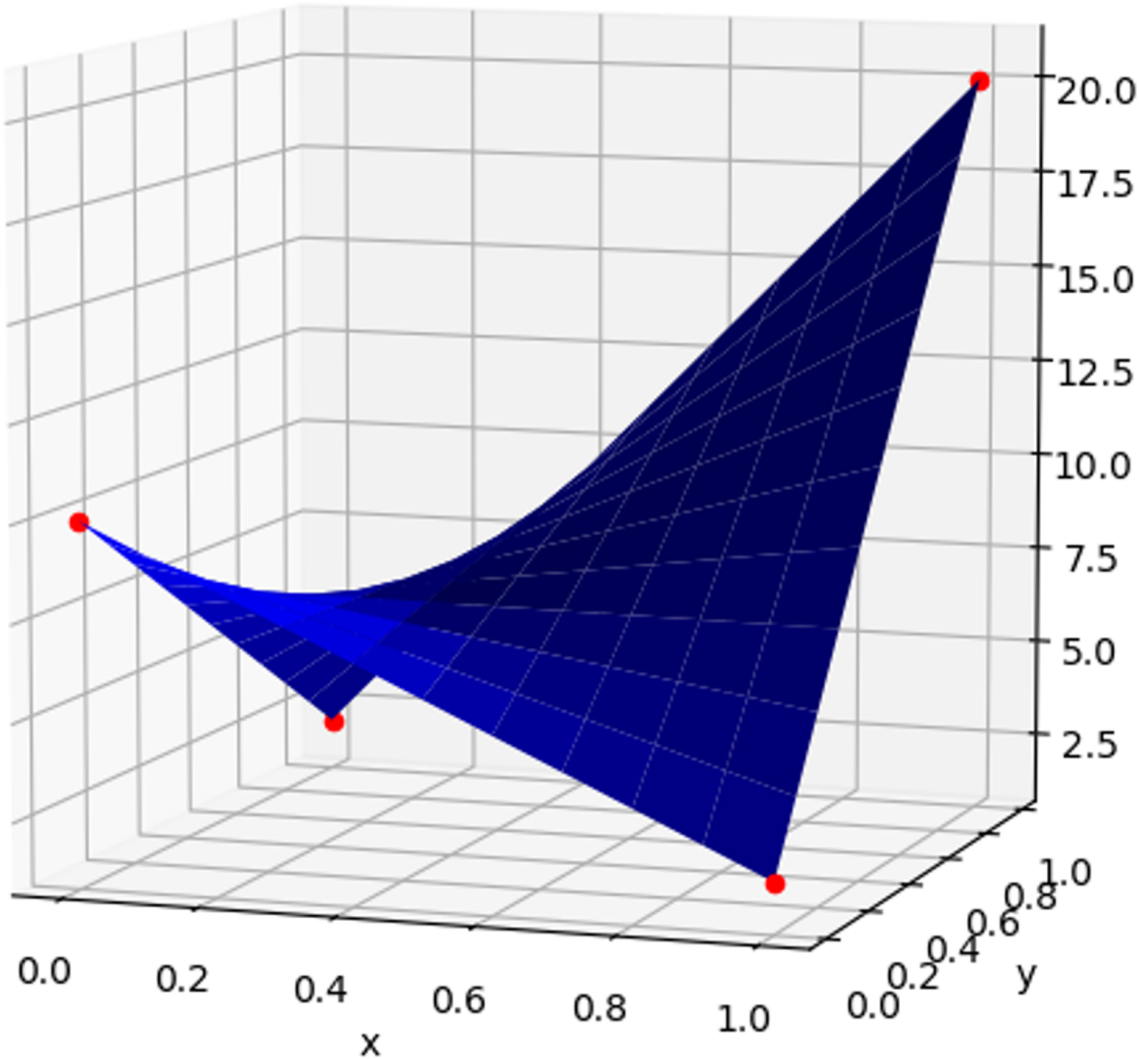
The values of the interpolants (red points) are used to calculate the bilinear interpolation (surface plot) using Equation (28).

An illustration of this is provided in Fig. 6.

Despite its name, as seen above, the resulting interpolant is not linear; rather, it is the sum of products of linear functions in *x* and *y*, respectively. Hence, the whole function is piecewise quadratic and may not be differentiable at the cell edges. Furthermore, the constrained optimization problem is transformed into an unconstrained problem using a penalty method. The penalty term in (27) includes a characteristic function, which is binary and not differentiable. In view of this, it is fitting to use optimization methods that do not rely on gradients. In this work, we explore the use of derivative-free metaheuristic algorithms in solving the minimization problem.

Metaheuristic algorithms have gained popularity because they only rely on function evaluations and have the capability of obtaining the global minimizer (Yang, 2014). It was shown in Ferrolino et al. (2020) how PSO was effective in solving the tsunami location problem without depth constraint. We adopt the same algorithm for our problem formulation.

**Figure 7:**
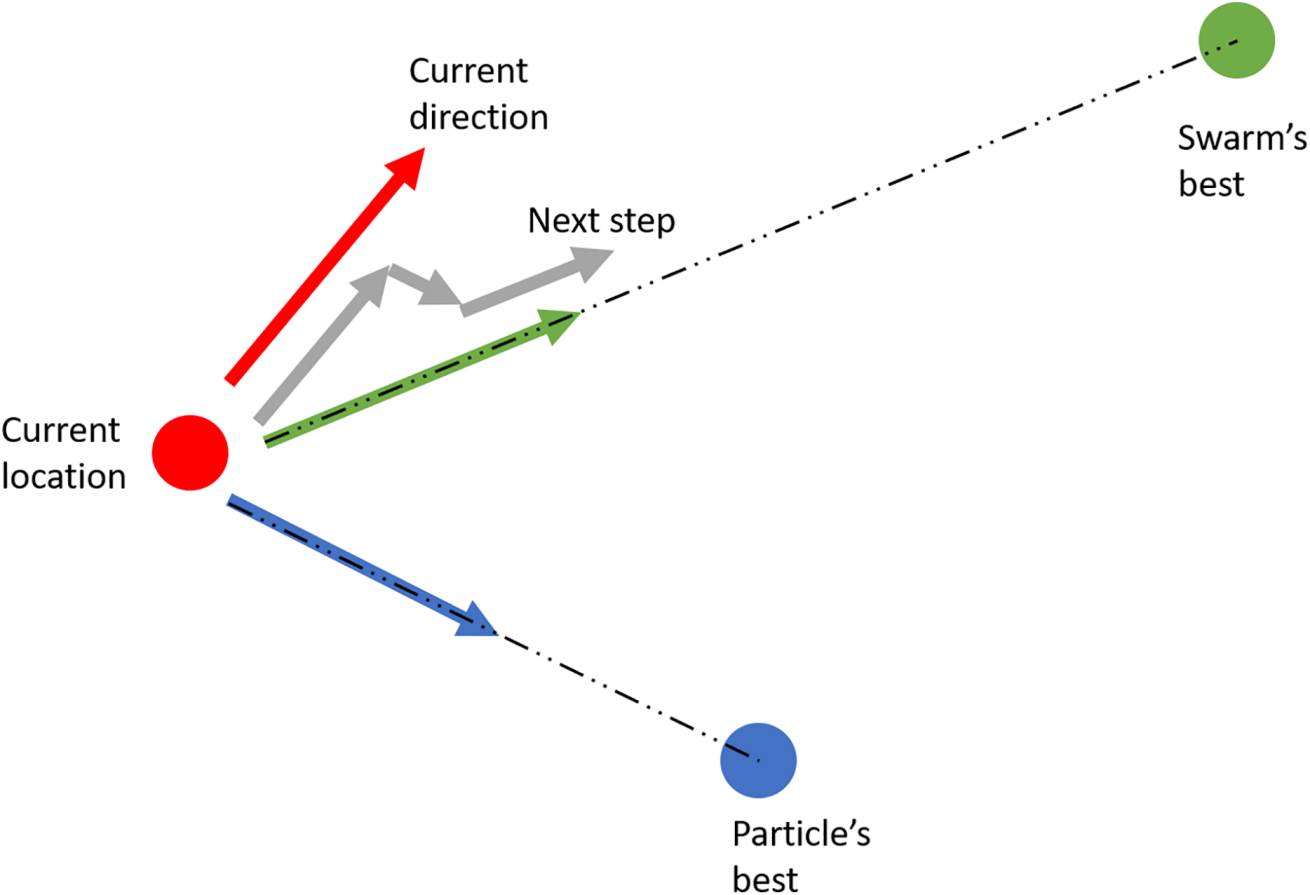
Illustration of the particle swarm optimization.

The PSO algorithm starts by randomly generating a swarm of solution candidates (particles) with their initial velocity vector. Each particle then move around the search-space according to three parameters: its velocity vector, the vector pointing at its own best-known position, and the vector pointing at the swarm’s best-known solution. For each iteration, the step that each particle takes is a linear combination of these three vectors (see Fig. 7), expecting that the swarm eventually gathers around one point—the optimal solution.

The pseudocode of the algorithm is presented in Algorithm 1. The algorithm starts by generating *swarm*_*size*_ particles randomly in the search space *D*, and each their own initial velocity vector **v**. They all then follow a series of instructions based on computations provided in Algorithm 1, where the vectors **p** (each particle’s best known location) and **g** (the entire swarm’s best known location) is updated. This process terminates when either the maximum number of iterations (*max*_*iter*_) is reached, or the change in the objective value *f* is less than *min*_*func*_. In this study, the function *f* is the one presented in Eq. (26); and the value of each hyperparameters are *swarm*_*size*_ *= 100, max*_*iter*_ *= 100, min*_*func*_ *= 10*^*−8*^, *ω = ϕ*_*p*_ *= ϕ*_*g*_ *= 0.5*.

**Algorithm 1:** Particle swarm optimization.

**input**: *D* (search space), *f* (objective function)
**output**: *g* (swarm’s best known location)
**hyperparameters:** *swarm*_*size*_, *max*_*iter*_, *min*_*func*_, *ω*, *ϕ*_*p*_, *ϕg*
**begin**
**let** $swarm \leftarrow generateSwarm(swar{m_{size}})$;
**for each** *particle* ∈ *swarm* **do** set its location as the best known position;
**let** $g \leftarrow arg{\kern 1pt} mi{n_p}f(p)$ *p*: each particle’s best known position
**for** $i \leftarrow 1$ **to** *max*_*iter*_ **do**
**for** *particle* ∈ *swarm* **do**
**let** *x*, *v*, *p* as the particle’s position, current velocity, best known position;
**let** ${r_p},{r_g} \leftarrow random(0,1)$;
$v \leftarrow \omega v + {r_p}{\phi _p}(p - x) + {r_g}{\phi _g}(g - x)$;
$x \leftarrow x + v$;
**if** *f*(*x*) < *f*(*p*) **then ** $p \leftarrow x$;
**end**
$g \leftarrow arg{\kern 1pt} mi{n_p}f(p)$;
**end**
**end**

More detailed discussions of this method may be found in Kennedy & Eberhart (1995); Shi & Eberhart (1998), Mezura-Montes & Coello (2011). Our implementation of the PSO algorithm made use of the built-in command *PySwarms*, an extensible research toolkit for particle swarm optimization in *Python*.

## Results

### Benchmark test and validation of SWE

In order to get an idea of the numerical model’s robustness and accuracy, some benchmark tests are performed. The model is considered using different types of initial conditions, boundary conditions, and bottom profiles. These simulations test the performance of the SWE.

#### 1D standing wave on a flat bottom

A standing wave, simply put, is a wave that does not travel (horizontally). Rather, it is an oscillating wave fixed in space. At any given point in space, the peak amplitude of wave oscillations on a standing wave is constant. This phenomenon can be observed on the surface of a liquid in a vibrating container.

**Figure 8:**
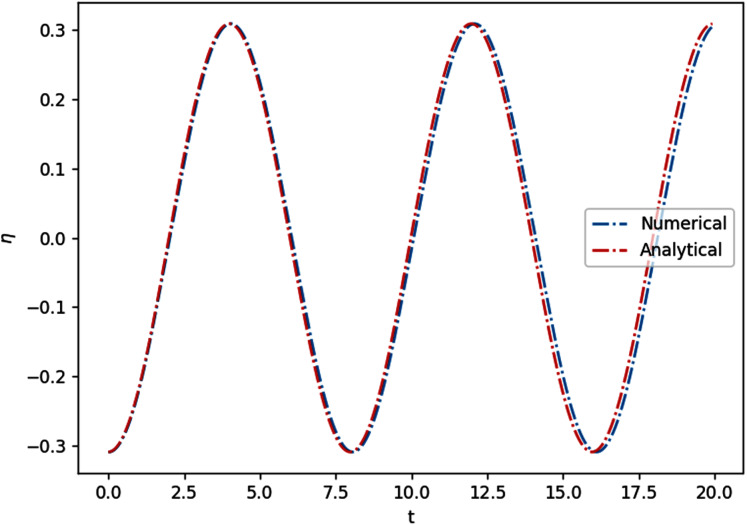
Comparison between numerical and analytical solutions of the 1D standing wave on a flat bottom, at x = 15 m.

To produce a standing wave simulation in one dimension, we set up a container of length *L*, constant still water depth *d*; give it initial conditions $\eta (x,0) = \cos (\displaystyle{{\pi x} \over L})$, *u*(*x*, 0) = 0 and wall boundary conditions *u*(0, *t*) = *u*(*L*, *t*) = 0. The result of the simulation on a flat bottom with length *L* = 25 m and depth of 4 m, using *Δx* = 0.2 m, is shown in Fig. 8. The wave seemingly does not travel horizontally, as a result of interference between two waves traveling in opposite directions. The water level at any point *c* oscillates in time with amplitude |*η*(*c*, 0)|. The analytical solution to this phenomenon is equivalent to that of a string of infinite length with some wave number *k* and angular frequency *ω*. In linear and non-dispersive case here, these numbers are just $k = \displaystyle{\pi \over L}$ and $\omega = k\sqrt {gd}$, which gives

(29) $$\eta (x,t) = \cos \left( {\displaystyle{\pi \over L}x} \right)\cos \left( {\displaystyle{\pi \over L}\sqrt {gd} {\kern 1pt} t} \right).$$

Simulating this at *Δx* = 0.2 m, *Δt* = 0.032 s for 20 s gives a mean absolute error of 0.015 m. To simplify things, all simulations below assume hard wall boundary conditions and no current, unless stated otherwise.

#### 1D wave run-up on a sloping beach

The first nonlinear simulation is wave run-up, which refers to either the phenomenon or the measure of when an ocean wave reaches a beach or sea dike structure and rises above still water level. Obviously, as a wave approaches the beach structure, the water depth gets smaller relative to the amplitude, hence the advection term becomes more significant. Furthermore, in this simulation the wave interacts with the dry region, something that was not observed in the previous simulations.

**Figure 9:**
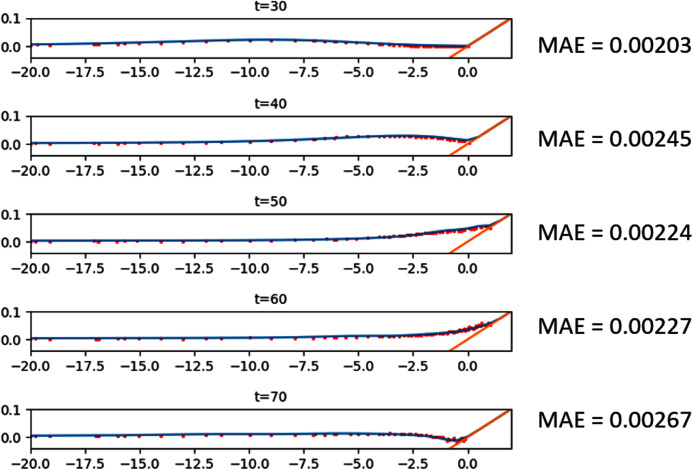
Comparison between Synolakis’ experiment and the numerical solution of the 1D wave run-up on a sloping beach.

This simulation is based on an experiment by Synolakis (1987) on a solitary wave that propagates through a single slope. The solitary wave centered at *x* = *x*_0_ has an initial surface profile $\eta (x,0) = \textstyle{H \over d}sec{h^2}\left( {\gamma \left( {x - \textstyle{L \over 4}} \right)} \right)$ with $\gamma = \sqrt {\textstyle{{3H} \over {4d}}}$. This way, similar to the step bottom simulation, the right traveling soliton eventually changes shape, this time as it climbs the sloping beach. This behavior shown in Fig. 9, where the simulation was done with *Δx* = 0.1 m and *Δt* = 0.016 s. Several gauges were placed during the experiment to record the wave height every 10 s. We then compared these records to the simulation. As seen in Fig. 9, the model was able to predict the experiment to a pretty high accuracy.

#### 1D dam break over a flat, dry bed

**Figure 10:**
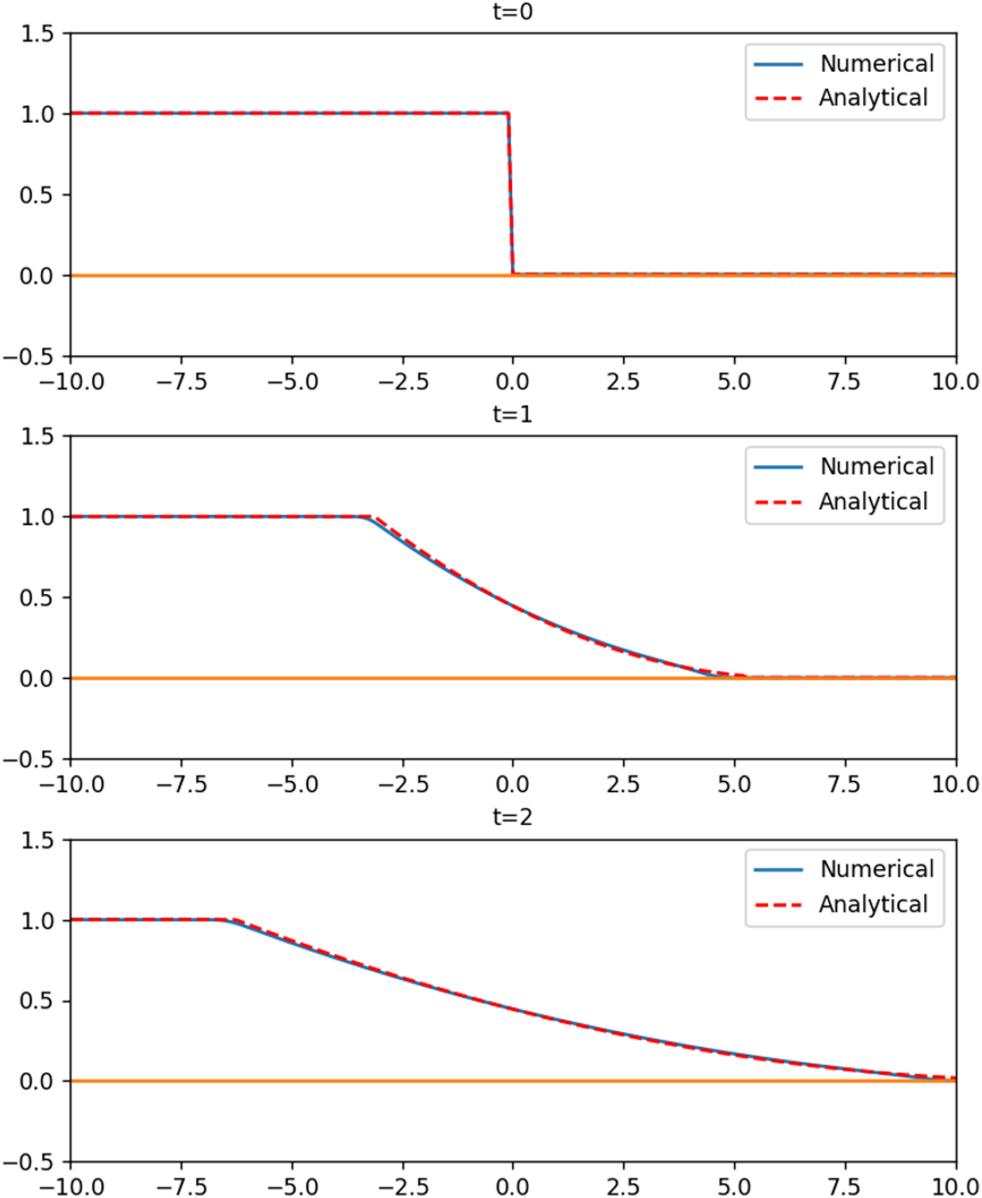
Comparison between the analytical and numerical solutions of the 1D dambreak over a flat, dry bed.

Dam break, as the name suggests, happens when the dam holding a reservoir breaks, causing a sudden rush of water flowing to the other side. The set up of this simulation is on a flat bed with 1-m-deep water on one side of the domain (reservoir), and completely dry on the other side:

(30) $$h(x,0) = \left\{ {\matrix{ {H,}  {x \lt  {x_0,}} \cr {0,}  {x \gt {x_0},}}  } \right.$$

where *x*_0_ represents the location of the barrier. Right after the simulation starts, the previously contained water will stream to the dry side and start to fill it, while the water level on the reservoir goes down. The result of the simulation on a 20 m-long, 1 m-deep bed with *Δx* = 0.1 m is provided in Fig. 10. To test the robustness of the numerical scheme, we compare it with the analytical solution given by Chanson (2008):

(31) $$h(x,t) = \left\{ {\matrix{ {H,}  {x \lt - ct,} \cr {\displaystyle{H \over 9}{{\left( {\displaystyle{{2 - x} \over {ct}}} \right)}^2},}  { - ct \le x \lt (2c - 1)t,} \cr {}  {} \cr } } \right.$$

where $c = \sqrt {gH}$. At *Δx* = 0.1 m, *Δt* = 0.008 s, the numerical scheme gives a mean absolute error of 0.0034 m.

#### 2D planar surface wave over a parabolic basin

So far the simulations were set on rectangular or trapezoidal shapes. To avoid bias due to the also rectangular shape of the grids, this simulation will be set on a parabolic basin. This way, not only do we have a curved bottom profile, all of the perimeter will be interacting with dry regions. The results of this simulation further confirms the performance of the wet-dry procedure.

The shape of the basin follows the parabolic function $d(x,y) = {d_0}\left( {1 - ({x^2} + {y^2})/{L^2}} \right)$ on an 8,000 m × 8,000 m domain, with *L*^2^ = 8,000 m^2^. The large parabolic shape of the domain is supposed to mimic that of a lake. We would like to see the motion of the water surface initialized by $\eta (x,y,0) = \textstyle{{2{a_0}{d_0}} \over L}(\textstyle{x \over L} - \textstyle{\xi \over {2L}})$. The analytical solution of the planar surface wave obtained by Thacker (1981) is

(32) $$\eta (x,y,t) = \displaystyle{{2\xi {d_0}} \over L}\bigg(\displaystyle{x \over L}\cos \omega t - \displaystyle{y \over L}\sin \omega t - \displaystyle{\xi \over {2L}}\bigg),\quad \omega = \displaystyle{1 \over L}\sqrt {2g{d_0}} .$$

**Figure 11:**
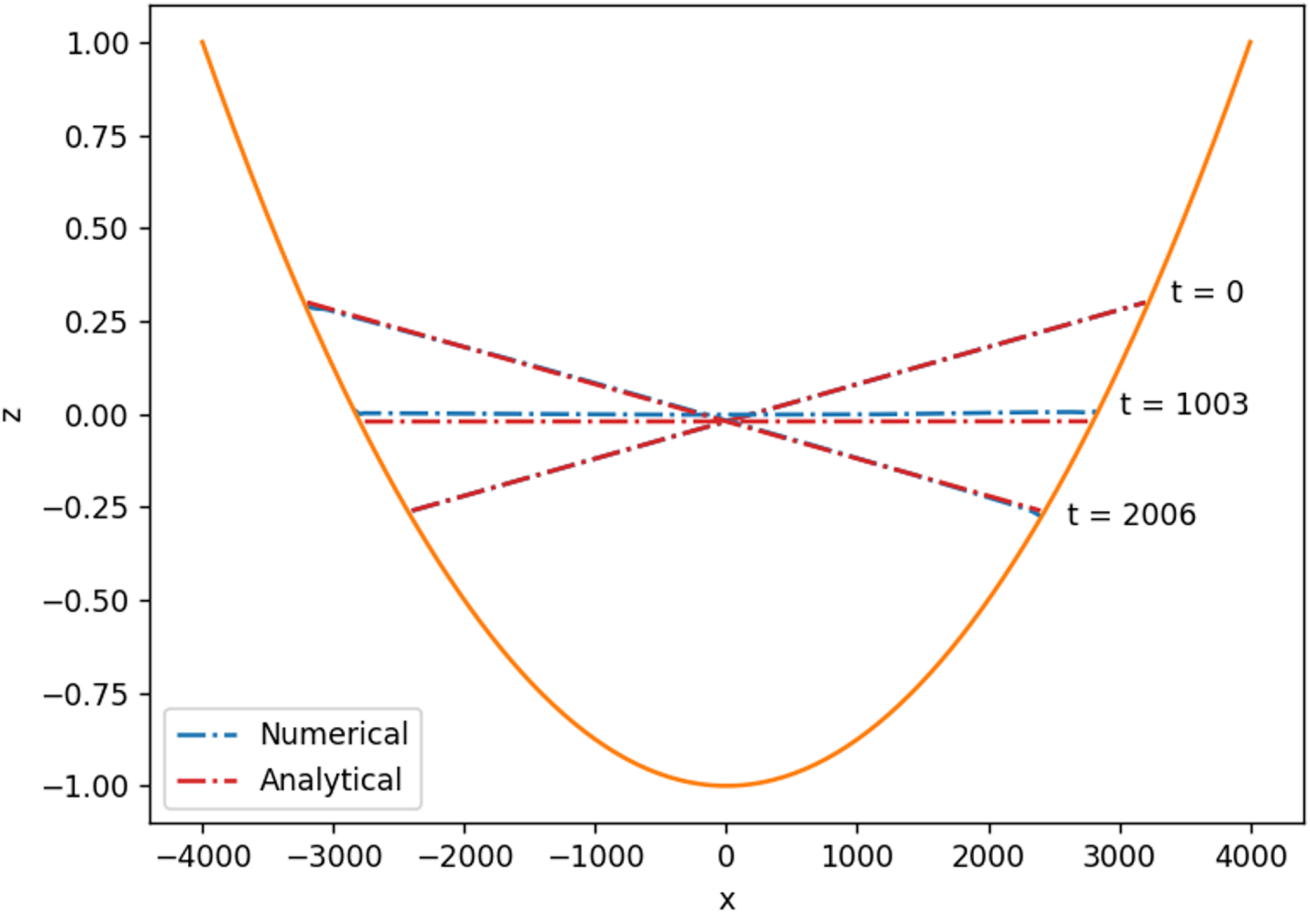
Cross section along y = 0 of the 2D planar surface wave over a parabolic basin.

The parameters used in the simulation were *d*_0_ = 1 m, *ξ* = 400 m using *Δx* = *Δy* = 40 m, and *Δt* = 4.5 s. As expected, the planar surface in the parabolic basin oscillates with period $T = 2\pi /\omega = 2\pi L/\sqrt {g{d_0}}$, which can be seen in Fig. 11. Quantitatively, the numerical solution agrees with the analytical solution with mean absolute error of 0.029 m.

### Validation with tsunami-related experiments

Finally, the performance of staggered finite volume scheme for the SWE will be tested on tsunami-related events. To do this, the model is tested on two experiments: “solitary wave on a conical island” and “tsunami run-up onto a complex 3D beach.”

#### Tsunami run-up onto a conical island

**Figure 12:**
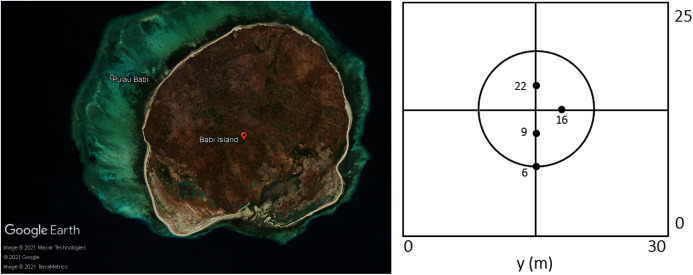
Satellite view of Babi Island (left); conical island experiment setup (right).

**Figure 13:**
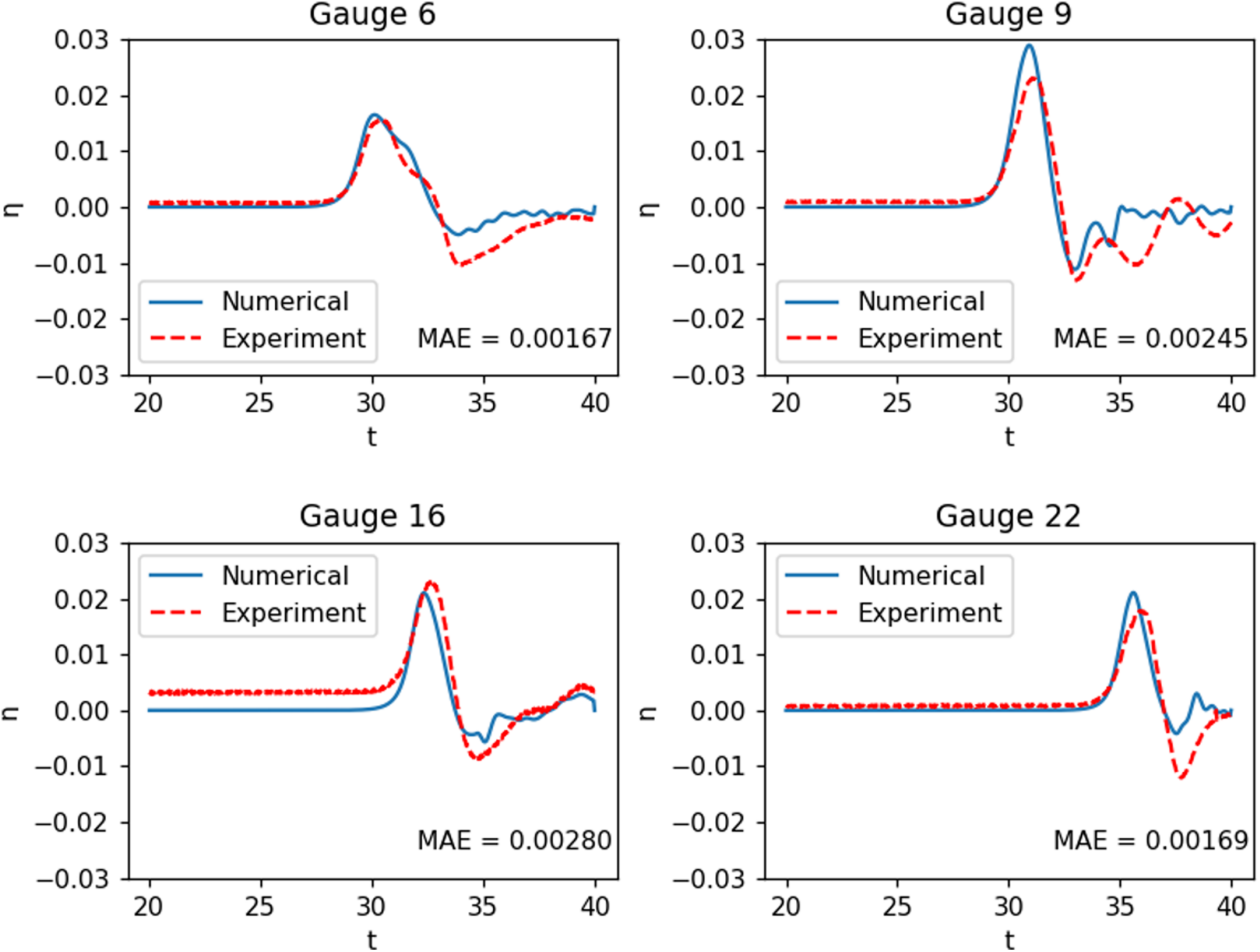
Comparison between the numerical solution of the tsunami run-up onto aconical island and data obtained from the laboratory experiment.

This experiment was done at the Coastal Engineering Research Center, Vicksburg, Mississippi, as part of a research (Briggs et al., 1995) on a tsunami that struck Babi Island, Indonesia in 1992. The shape of Babi Island is similar to a truncated cone, as seen in Fig. 12, and the experiment may serve as a benchmark test for fluid dynamic models in modeling interaction between waves and such structure. It was conducted on a 25 m × 30 m basin with a conical island at the center, and waves with an initial solitary wave-like profile were generated from one side of the tank. The resulting water surface elevations were measured by 22 gauges. Our numerical simulation was ran with *Δx* = *Δy* = 0.2 m. The results of the simulation were compared with data gathered at 4 of the 22 gauges: G6, G9, G16, and G22. As seen in Fig. 13, the model was able to replicate the experiment with great accuracy.

#### Tsunami run-up onto a complex 3D beach

**Figure 14:**
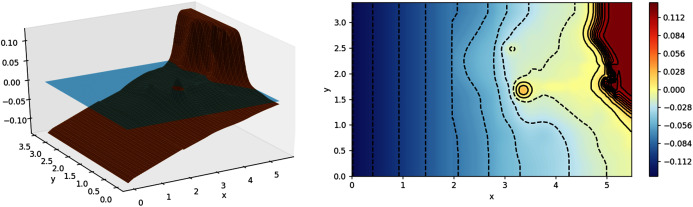
Monai laboratory model (left); contour of Monai laboratory model (right).

**Figure 15:**
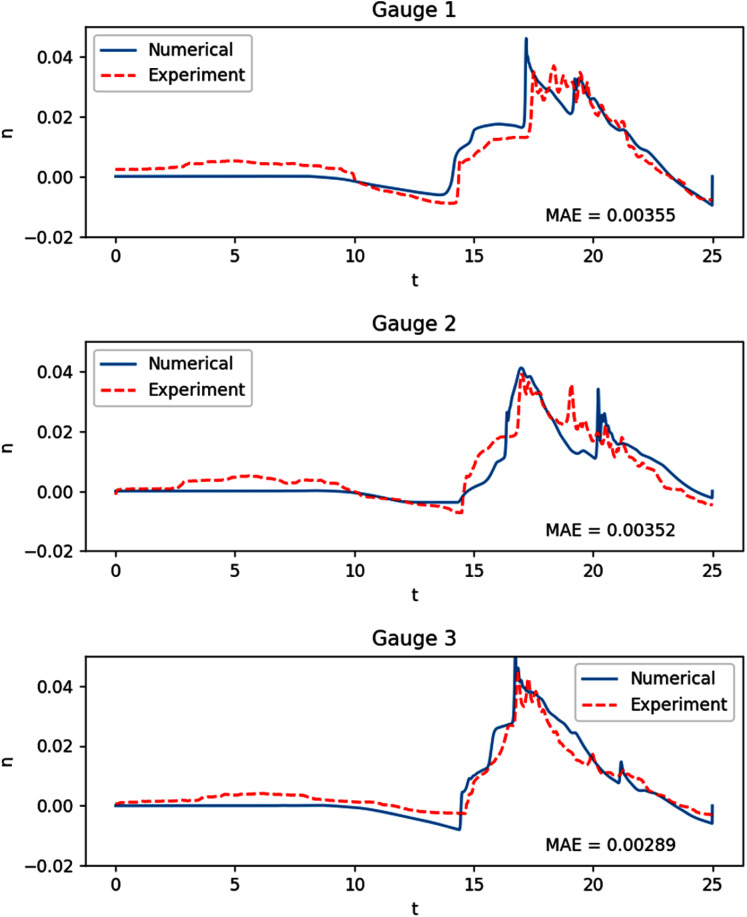
Comparison between the numerical solution of the tsunami run-up onto a complex 3D beach and data obtained from the laboratory experiment.

We then move on to an experiment with a much more complex bathymetry, similar to that found in nature. The experiment was based on a tsunami that struck Okushiri Island, Japan, with a really high current, that resulted in an extreme run up at the tip of a very narrow gulley within a small cove at Monai. It was done at the Central Research Institute for Electric Power Industry (CRIEPI) in Abiko, Japan on a 1:400 laboratory model of Monai (Liu, Yeh & Synolakis, 2008) and the setup in shown in Fig. 14. Just like the previous experiment, a series of waves were generated from the deeper side of the water. The results of our simulation, with *Δx* = *Δy* = 0.025 m, were compared with the recorded height on the three gauges placed during the experiment. As can be observed in Fig. 15, the model was able to replicate the experiment with high accuracy.

## Optimal placement of detection sensors

Having established the robustness of the shallow-water model, we now solve the optimization problem under different scenarios. This section is divided into three parts. First, we investigate the case when there is only one sensor available. In the next part, we look at how the first case compares with having multiple detection sensors. Finally, we implement the proposed method using data from the 2018 Palu tsunami incident.

### One detection sensor

#### Step bottom

**Figure 16:**
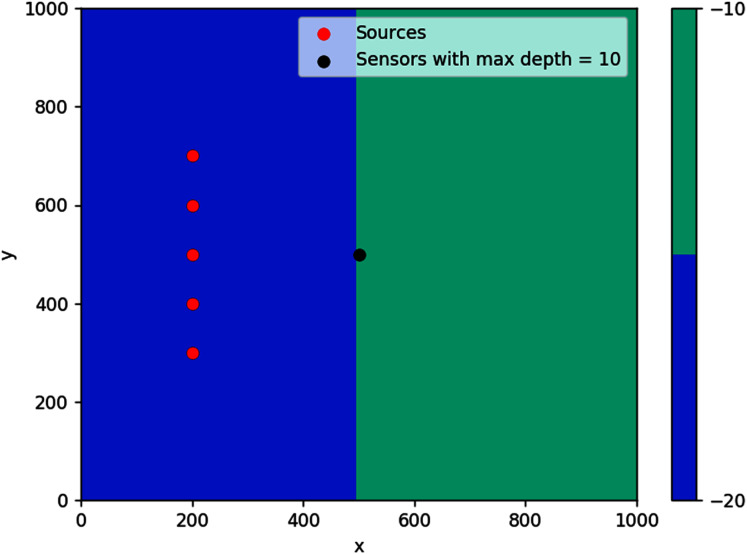
Optimal location of one detection sensor on a step bottom.

The first simulation was done on a step bathymetry of size 1,000 m × 1,000 m with *d* = 20 m for *x* < 500 m, and *d* = 10 m elsewhere. Waves were generated from five evenly spaced points along the line *x* = 200 m, as seen in Fig. 16. The feasible region for this problem is {(*x*, *y*): *d*(*x*, *y*) ≤ 10 m}, which is the whole right side of the domain.

The result obtained is pretty intuitive, that is, the optimal location of detection sensor in this case is right in the middle of the line where the domain changes depth. If located far up on the *y*-axis, it will take long for the sensor to detect waves coming from source points further down (and vice versa). If placed too far to the right, waves from any source point will take a longer time to reach the sensor.

#### Sloping bottom

**Figure 17:**
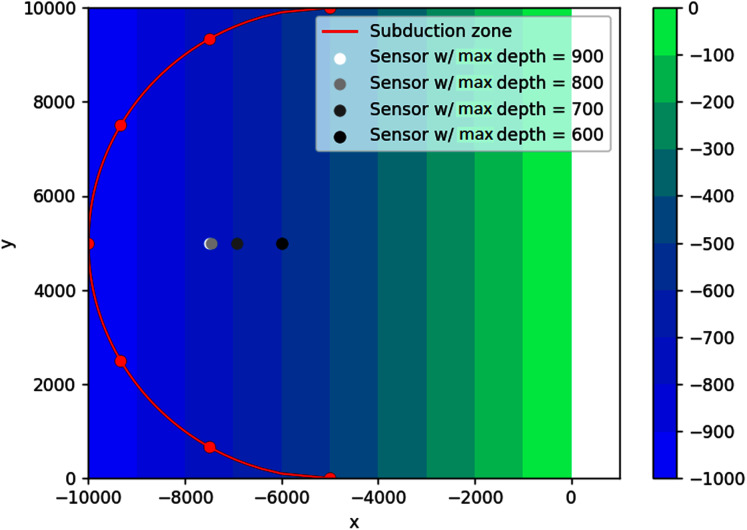
Optimal location of one detection sensor on a sloping bottom with varying depth constraints.

The next simulation was done on a sloping bathymetry of size 10,000 m × 10,000 m, with a slope of 0.1 and maximum depth of 1,000 m. This time the waves were generated through a flip-fault motion from six subduction zones in the shape of arcs of the semicircle $x + 5000 = - \sqrt {{{5000}^2} - {{(y - 5000)}^2}}$, as seen in Fig. 17. The feasible region for this problem is {(*x*, *y*): *d*(*x*, *y*) ≤ *d*_*max*_, *x* < −5,000}, with *d*_*max*_ = 900, 800, 700, 600 m. Since in this case the water depth gradually gets smaller as we approach the shoreline on the right side, it is interesting to investigate what happens if the depth constraints are varied.

As seen in Fig. 17, not all sensors go to their respective depth constraint. This is expected because the bottom topography is not flat. As predicted, the sensors obtained with varying depth constraints lie on the bisector of the semicircle. As the water depth increases, the location of the sensors move away from the shore and closer to the subduction zones. However, the sensor cannot get too close to the semicircle to guarantee detection time from all the possible sources. This can be seen on the obtained locations of sensors for depths 800 and 900, *i.e*., the two locations are almost identical. This is possible because the feasible region for *d*_*max*_ = 800 m is contained in the feasible region for *d*_*max*_ = 900 m.

### Multiple detection sensors

#### Step bottom

**Figure 18:**
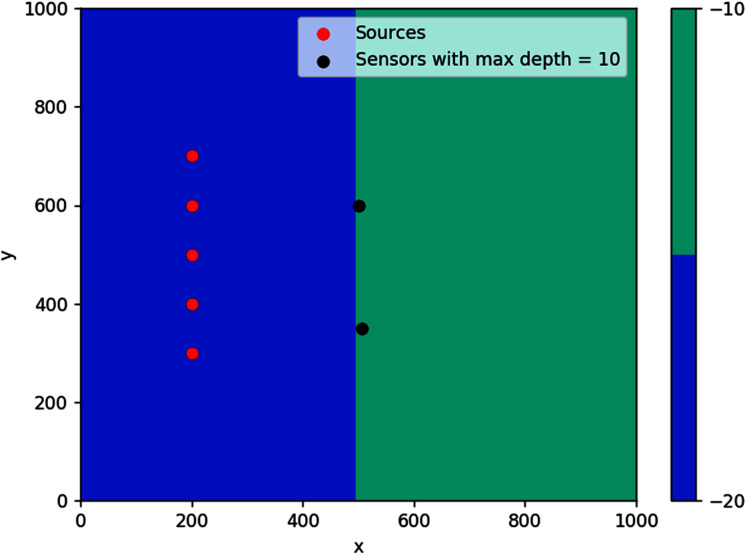
Optimal location of two detection sensors on a step bottom.

Using the same setup as the previous one, the PSO algorithm was run to solve the optimal location of two detection sensors. As seen in Fig. 18, the obtained locations of the sensors are on the boundary of the feasible region. This is expected because the sensors must be placed as close as possible to the source points and at the same time must satisfy the depth constraint. It also makes sense that neither sensor was located right in the middle, since one sensor can account for waves coming from the upper side while the other can account for waves coming from the lower side.

#### Sloping bottom

**Figure 19:**
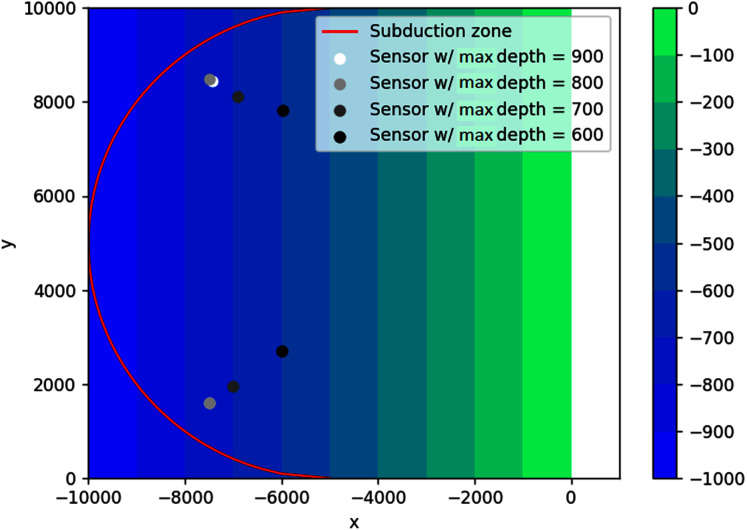
Optimal location of two detection sensors on a sloping bottom with varying depth constraints.

**Figure 20:**
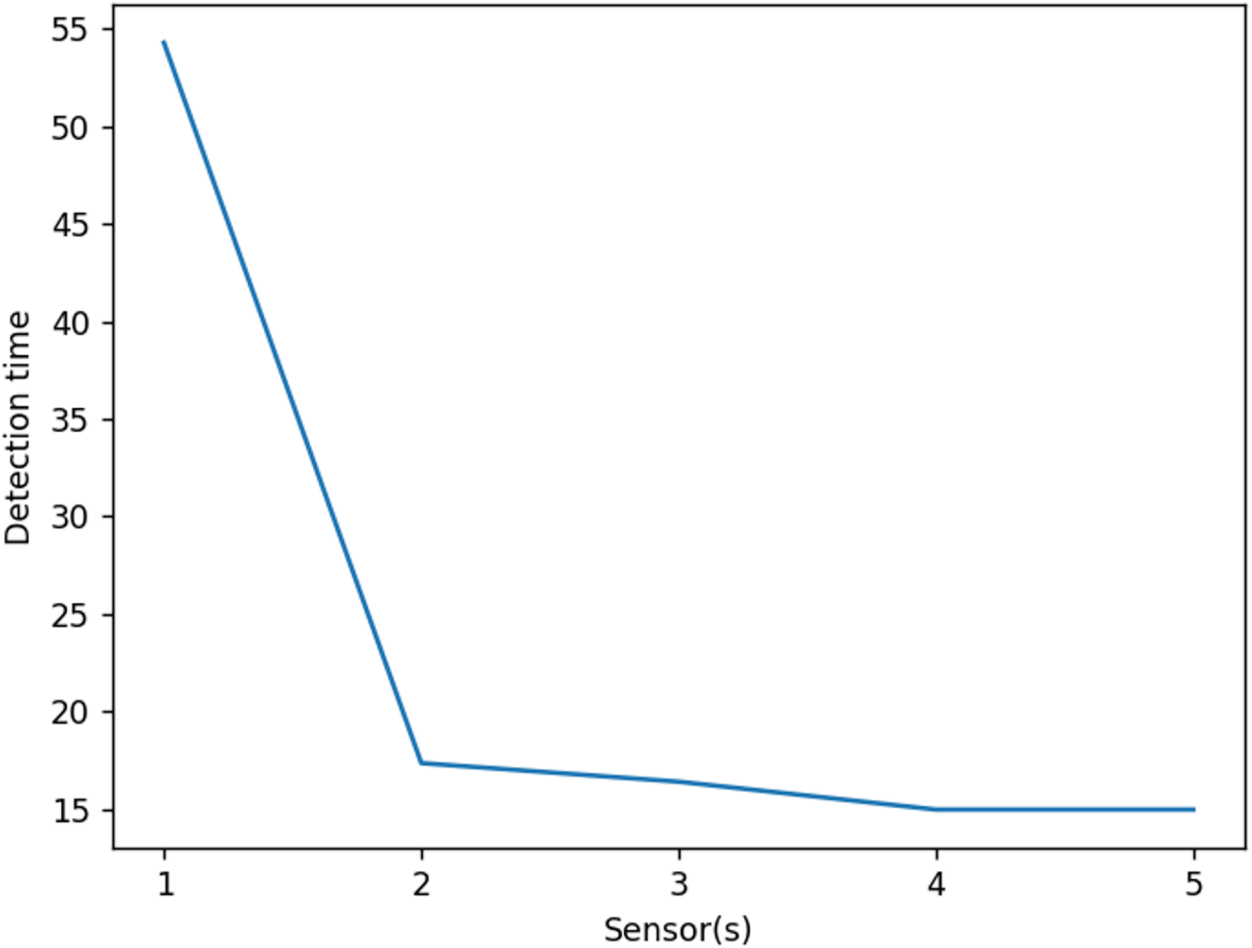
Effects of placing multiple detection sensors on wave detection time (*d_max_* = 700 m).

In this example, the optimal locations of sensors are more spread out to account for waves coming from different angles. The results are illustrated in Fig. 19. Obviously, increasing the number of sensors will always decrease the wave detection time. As shown in Fig. 20, using two sensors instead of one allows the waves to be detected about 37 s earlier or 68% faster. However, using three or more sensors would be less efficient, as this only decreases the detection time by 1 s or even less.

#### Sloping bottom with an island

**Figure 21:**
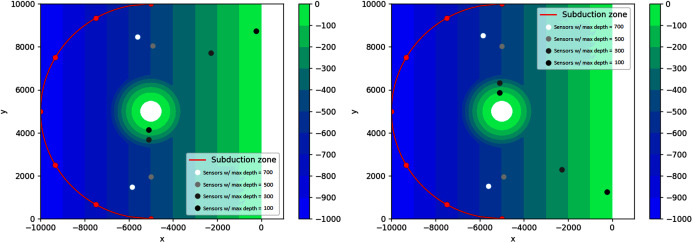
Two obtained solutions for the optimal placement of two sensors on asloping bottom with an island subject to varying depth constraints.

This simulation starts with the same setup as the sloping bottom simulation, only this time we introduce a small island in the middle of the domain. In this case, we expect to see interesting results on small depth constraints since there are two “shallow” areas here: the center island and the coastline on the right. It can be seen in Fig. 21 that sensors with depth constraints of 500, 700 m are not really affected by the island. However for maximum depth of 300 and 100 m, the pair of sensors are spread apart, with one approaching the island and the other getting closer to the coastline on the right. Because the domain is symmetric with respect to the line *y* = 5,000 m, two solutions are obtained. This is illustrated in Fig. 21. This configuration could not be tackled in Ferrolino et al. (2020) because the presence of an island in the simulation requires the use of the nonlinear SWE with wet-dry procedure.

The simulations we have presented so far were formulated so that the optimal solutions can be verified based on the geometry of the subduction zone and the domain. The obtained results using PSO yield solutions that agree with the expected geometric solutions. These tests were done to check that the method works. In a real-world scenario, the optimal placement of sensors is not trivial because the subduction zones do not necessarily follow a geometric pattern. Additionally, the bottom topography of oceans is uneven, making the computation of travel time more complicated. Because we use the 2D nonlinear SWE in simulating tsunami waves, our proposed method can handle complex bathymetric profiles and arbitrary tsunami sources. In our following and last example, we apply our tsunami sensor detection model to one of the more recent tsunami incidents: the 2018 Palu tsunami.

### Application on real events: 2018 Palu tsunami

On 28 September 2018, Central Sulawesi was struck by a 7.5-Richter-scale earthquake, which was followed by a tsunami sweeping through the city of Palu.

**Figure 22:**
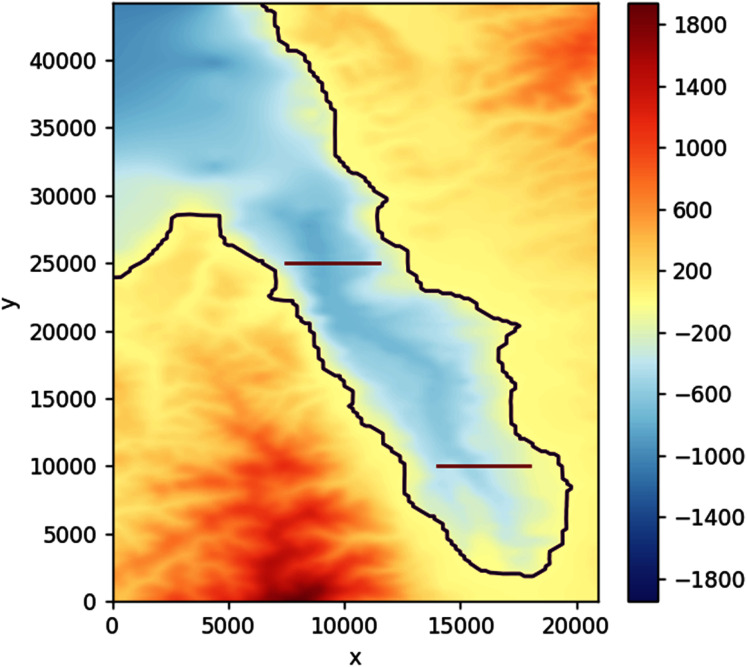
Filled contour plot of Palu Bay. The possible sources of the tsunami are landslides along the lines (in red) as proposed by Heidarzadeh, Muhari & Wijanarto (2018).

To simulate the tsunami, we use a gridded bathymetry of Palu Bay provided by BATNAS Indonesia, with a grid size of 185 m × 185 m. Landslides are generated from two subduction zones that were proposed by Heidarzadeh, Muhari & Wijanarto (2018) using backward tsunami ray tracing, see Fig. 22.

**Figure 23:**
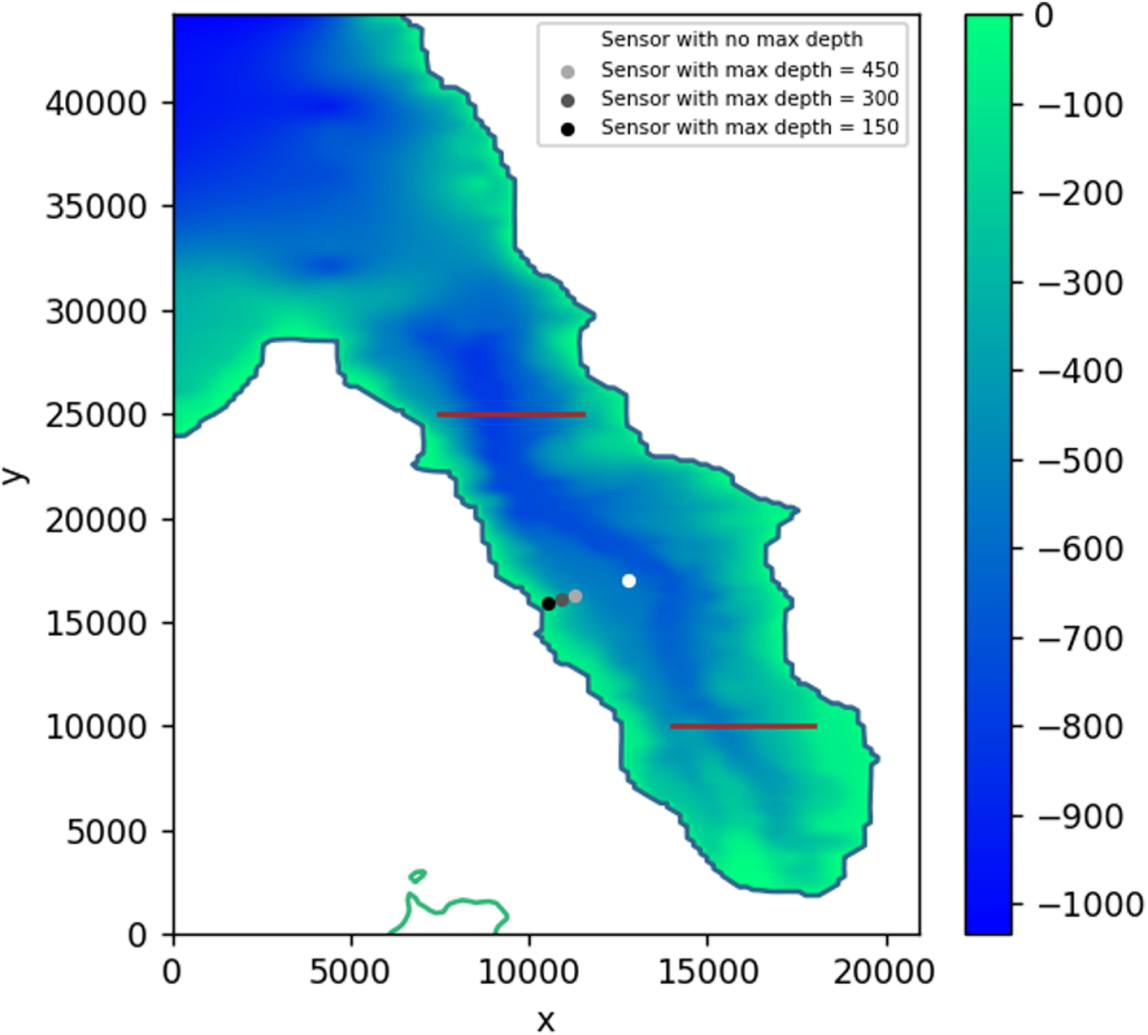
Optimal location of one detection sensor, with varying depth constraints, based on 2018 Palu tsunami incident.

Solving the optimization problem using PSO on this simulation without constraints found the optimal sensor placement at depth *d* = 595.48 m with coordinates: longitude = 119.81583, latitude = −0.74583. As can be seen in Fig. 23, this location is approximately in the middle of the region bounded by the two subduction zones. Adding some more depth constraints, from 450 to 150 m, consistently moved the sensor closer to the shore.

Assuming no constraints, placing the detection sensor according to this simulation would allow the waves coming from either side to be detected in just about 93 s. This shaves off up to 240 s compared to the case when the sensor was placed more to the south, closer to Talise Beach, which would then take either 113 s or 333 s depending on the source. If a depth constraint of 150 m is applied, the optimally located sensor would still be able to detect the tsunami in about 106 s, saving up to 227 s.

## Conclusion

To conclude, this paper has accomplished two significant results. First, we have derived a robust and accurate numerical shallow water model (SWE) for simulating water waves in different situations. The wet-dry procedure that are included in the calculation can simulate wave propagates over a dry area. Secondly, we have integrated this shallow water model in conjunction with the meta-heuristic PSO algorithm to solve various optimization problems of placing tsunami detection sensors, including the 2018 Palu tsunami incident. The latter findings proved that strategic placement of detection sensors for tsunami warning systems can drastically improve detection time, giving the responsible parties more time to evacuate the citizens.

In the previous work by Ferrolino et al., 2020, the tsunami sensors can be placed anywhere in the water domain. Their results yield the placement of sensors near the subduction zones for faster detection time. However, subduction zones are usually located in the deep ocean, making the placement of sensors costly. In the Philippines, tsunami warning systems are installed near the shores. Because of this depth constraint, we modify the optimization problem proposed in Ferrolino et al. (2020). We use a penalty method to solve the constrained minimization problem, which is a generalization of the tsunami detection problem presented in Ferrolino et al. (2020). The arising minimization problem is solved using PSO, a robust and easy-to-implement global optimization algorithm.

The benchmark tests for the tsunami sensors location problem are formulated so that the optimal solution can be inferred geometrically. This way, we can assess if the obtained solution by PSO is correct. Our numerical results agree with the expected solution. However, it is still interesting to know what happens if other numerical optimization algorithms are used. A comparative analysis of recent metaheuristic optimization algorithms applied to tsunami sensor detection problems merits a separate study.

In our test simulations, the possible tsunami sources are evenly placed along the subduction zone. However, in reality, some places in the subduction zone may be more tectonically active than others. This is a limitation of the current study. For future research, one can use time-series data of earthquakes within the subduction zone and use a clustering algorithm to identify coordinates along the zone that will most likely be a tsunami source.

## Additional Information and Declarations

### Data Availability

The following information was supplied regarding data availability: The data used in benchmark tests are available at the NOAA website: 1. Conical Island https://nctr.pmel.noaa.gov/benchmark/Laboratory/Laboratory_ConicalIsland/index.html 2. Monai Valley https://nctr.pmel.noaa.gov/benchmark/Laboratory/Laboratory_MonaiValley/ The bathymetry data for Palu simulation are available in http://batnas.big.go.id/pencarian?kategori=Batimetri&keyword=palu/ The Python code used to run the simulations are available at GitHub: https://github.com/raynaldilalang/SWE-PSO.
